# Clinical Characteristics, Antimicrobial Susceptibility, Treatment, and Outcomes of *Achromobacter* Bacteremia: A 20-Year Retrospective Cohort Study

**DOI:** 10.3390/antibiotics15100953

**Published:** 2026-09-25

**Authors:** Tawin Khaimook, Pisud Siripaitoon, Sarunyou Chusri, Narongdet Kositpantawong, Siripen Kanchanasuwan, Sorawit Chittrakarn, Nattapat Sangkakul, Nonthanat Tongsengkee

**Affiliations:** Division of Infectious Diseases, Department of Internal Medicine, Faculty of Medicine, Prince of Songkla University, Songkhla 90110, Thailand; grippen45@gmail.com (P.S.); sarunyouchusri@hotmail.com (S.C.); narongdet032@gmail.com (N.K.); kaymed29@yahoo.com (S.K.); sorawit.c@psu.ac.th (S.C.); wignattapat@hotmail.co.th (N.S.); tnonthanat@gmail.com (N.T.)

**Keywords:** *Achromobacter*, bacteremia, antimicrobial resistance, antimicrobial susceptibility testing, carbapenem, empiric therapy, mortality

## Abstract

Background/Objectives: *Achromobacter* bacteremia is uncommon, difficult to treat and lacks historically consistent organism-specific susceptibility criteria. We described its clinical characteristics, antimicrobial exposure, susceptibility patterns, treatment, and outcomes over 20 years. Methods: We retrospectively reviewed adults with *Achromobacter*-positive blood cultures at a Thai tertiary-care hospital during 2006–2025. The first eligible episode per patient was used for patient-level analyses. Historical susceptibility categories were retained as reported. Available matched blood-isolate minimum inhibitory concentrations (MICs) were retrospectively interpreted using Clinical and Laboratory Standards Institute (CLSI) M100, 36th edition. Results: Forty-nine eligible episodes occurred in 47 patients, of which 43/47 (91.5%) were nosocomial. Prior systemic antibiotics and carbapenems had been received by 43/45 (95.6%) and 26/45 (57.8%) patients, respectively. Prior carbapenem exposure was associated with meropenem non-susceptibility (50.0% vs. 11.1%; odds ratio (OR) 7.64, *p* = 0.010). Thirty-day mortality was 23/46 (50.0%). Non-survivors had higher median National Early Warning Score (NEWS) than survivors (6 vs. 3; *p* = 0.005). Mortality was lower among patients receiving active than inactive empiric therapy (39.4% vs. 76.9%; OR 0.20, *p* = 0.047), but the association attenuated after adjustment for vasopressor use. Historical disk and contemporary MIC categories were not fully concordant in the small matched subset (*n* = 7). Conclusions: *Achromobacter* bacteremia occurred predominantly as a nosocomial infection in heavily antibiotic-exposed patients and was associated with high mortality. Prior carbapenem exposure was associated with meropenem non-susceptibility, while mortality was closely related to acute illness severity. Historical susceptibility results should be interpreted in the context of the testing method and breakpoint framework.

## 1. Introduction

*Achromobacter* species are aerobic, non-fermenting Gram-negative bacilli increasingly recognized as opportunistic pathogens in patients with repeated healthcare exposure, immunocompromising conditions, indwelling devices, and structural lung disease. Although respiratory isolation in cystic fibrosis has received substantial attention, invasive infection outside cystic fibrosis remains uncommon and incompletely characterized [1,2]. Bloodstream infection has been described particularly in hospitalized and immunocompromised patients, with vascular catheters and primary bacteremia among the reported sources [2,3].

Treatment is complicated by both intrinsic and acquired resistance. *Achromobacter* spp. are intrinsically resistant to several antimicrobial classes, including many cephalosporins, aztreonam, and aminoglycosides, while acquired carbapenem resistance may be mediated by multidrug efflux systems and metallo-β-lactamases [1]. Consequently, piperacillin-tazobactam, carbapenems, trimethoprim-sulfamethoxazole, and ceftazidime have historically been among the most commonly considered agents, but susceptibility varies substantially across settings and isolates [1,2,3]. Newer agents such as cefiderocol have been used as salvage therapy, although clinical evidence remains limited to small observational series [4].

Antimicrobial susceptibility testing (AST) is an additional challenge. Historical studies used heterogeneous methods and interpretive criteria, and disk diffusion breakpoints specific to *Achromobacter* were not available for many years [5]. More recently, comparative minimum inhibitory concentration (MIC) and disk-diffusion studies have supported *Achromobacter*-specific tentative breakpoints, highlighting limitations of applying generic non-Enterobacterales criteria to this genus [6].

In our center, *Achromobacter* bacteremia appeared to be encountered more frequently in recent years, often after extensive exposure to broad-spectrum antibiotics. We therefore conducted a 20-year retrospective cohort study to describe temporal trends, clinical characteristics, antimicrobial exposure, AST patterns, treatment, and outcomes. We additionally explored the relationship between prior carbapenem exposure and meropenem non-susceptibility, the association between active empiric therapy and 30-day mortality, and categorical agreement between historical disk-based results and contemporary MIC reinterpretation in an available subset.

## 2. Results

### 2.1. Study Population and Temporal Distribution

A total of 49 eligible bacteremia episodes were identified in 47 unique adult patients (Figure 1). The first eligible episode per patient was used for the primary patient-level clinical and mortality analyses, while all 49 episodes were retained for temporal descriptive analyses. The median age was 60.0 years (interquartile range (IQR) 51.5–71.0), and 33/47 (70.2%) patients were male. Malignancy was the most common major underlying condition, affecting 22/47 (46.8%) patients, including 17/47 (36.2%) with solid malignancy and 5/47 (10.6%) with hematologic malignancy. Other notable comorbidities included hypertension (11/47, 23.4%), chronic cardiac disease (8/47, 17.0%), diabetes mellitus (6/47, 12.8%), chronic kidney disease/end-stage renal disease (6/47, 12.8%), and cerebrovascular disease (5/47, 10.6%). Most episodes were nosocomial (43/47, 91.5%), with a median interval of 15 days (IQR 5.0–49.5) from admission to the index blood culture (T0).

Of the 49 eligible bacteremia episodes identified during 2006–2025, 22 (44.9%) occurred during 2023–2025 (Figure 2).

### 2.2. Baseline Severity, Vital Signs, Laboratory Findings, and Mortality

Thirty-day vital status was available for 46/47 patients; 23/46 (50.0%) died. Any malignancy was more frequent among patients who died within 30 days than among survivors (65.2% vs. 26.1%; *p* = 0.017). Patients who died had greater physiologic and laboratory derangement at T0, including lower blood pressure, higher respiratory rate and National Early Warning Score (NEWS), more frequent vasopressor use, thrombocytopenia, higher bilirubin concentrations, hypoalbuminemia, and elevated lactate. Median NEWS was 6.0 (IQR 3.0–8.0) among deaths versus 3.0 (IQR 1.0–5.0) among survivors (*p* = 0.005). Baseline characteristics are summarized in Table 1.

### 2.3. Infection Characteristics, Antimicrobial Pressure, and Treatment

Antecedent systemic antimicrobial exposure within 90 days was nearly universal, while prior carbapenem and colistin exposure were also common (Table 1). Primary bacteremia was the most common source category (26/47, 55.3%), followed by catheter-related bloodstream infection (CRBSI) (9/47, 19.1%), ventilator-associated pneumonia (VAP) (4/47, 8.5%), urinary tract infection (UTI) (3/47, 6.4%), prosthetic valve endocarditis (1/47, 2.1%), and other or mixed sources (4/47, 8.5%). Polymicrobial bacteremia occurred in 13/47 (27.7%) patients (Table 2).

Nearly half of patients were already receiving systemic antimicrobial therapy at the time of the index blood culture, including frequent exposure to carbapenems and/or colistin. In some patients, bacteremia occurred while receiving at least one agent subsequently reported as susceptible (Table 2). Active empiric therapy, defined using the contemporaneously reported historical AST result, was given to 34/47 (72.3%) patients. Directed combination therapy was used in 5/47 (10.6%), and the median treatment duration was 10 days (IQR 5–14). Median hospital length of stay was 44 days (IQR 16–76.5) overall and did not differ significantly between 30-day non-survivors and survivors (41 vs. 52 days; *p* = 0.170). Follow-up blood cultures were obtained in 34/47 (72.3%) patients, with persistent *Achromobacter* bacteremia documented in 8/34 (23.5%).

### 2.4. Historical Susceptibility and Automated MIC Subset

Historical categorical susceptibility was highest for piperacillin-tazobactam and ceftazidime, lower for carbapenems and trimethoprim-sulfamethoxazole, and poor for ciprofloxacin (Table 3). Colistin results are presented only as historical laboratory-reported categories because no applicable contemporary susceptible breakpoint was available.

Among 44 patients with both prior-carbapenem exposure data and interpretable meropenem susceptibility results, meropenem non-susceptibility was observed in 13/26 (50.0%) patients with prior carbapenem exposure versus 2/18 (11.1%) without prior exposure (odds ratio (OR) 7.64, 95% confidence interval (CI) 1.35–81.83; *p* = 0.010).

Seven laboratory-confirmed paired blood-isolate MIC panels were included in the primary comparison. Meropenem showed complete categorical agreement, whereas piperacillin-tazobactam and ceftazidime showed the greatest discordance, including historical disk-susceptible isolates reclassified as intermediate or resistant by contemporary MIC criteria (Table 4). Alternative Clinical and Laboratory Standards Institute (CLSI) and European Committee on Antimicrobial Susceptibility Testing (EUCAST) breakpoint frameworks changed selected categorical assignments but did not alter this overall pattern. De-identified isolate-level MIC results and sensitivity analyses using alternative breakpoint frameworks are provided in Tables S1 and S2.

### 2.5. Factors Associated with 30-Day Mortality

In univariable analyses, higher odds of 30-day mortality were associated with prior carbapenem exposure within 90 days (OR 4.43, 95% CI 1.09–20.31; *p* = 0.032), ongoing systemic antimicrobial therapy at T0 (OR 4.14, 95% CI 1.07–17.73; *p* = 0.038), arterial-line use at T0 (OR 4.99, 95% CI 1.15–26.69; *p* = 0.029), vasopressor use at T0 (OR 12.81, 95% CI 2.27–138.37; *p* = 0.001), higher NEWS (OR 1.39 per point, 95% CI 1.09–1.76; *p* = 0.007), and malignancy (OR 5.10, 95% CI 1.28–23.00; *p* = 0.017). Active empiric therapy (OR 0.20, 95% CI 0.03–0.98; *p* = 0.047), higher systolic blood pressure (OR 0.72 per 10 mmHg, 95% CI 0.55–0.94; *p* = 0.016), and higher serum albumin (OR 0.14 per 1 g/dL, 95% CI 0.04–0.53; *p* = 0.004) were associated with lower odds of 30-day mortality.

In the parsimonious multivariable model including active empiric therapy and vasopressor use at T0, vasopressor use remained independently associated with higher mortality (adjusted odds ratio (aOR) 10.43, 95% CI 1.88–57.93; *p* = 0.007), whereas the association between active empiric therapy and mortality was attenuated and was no longer statistically significant (aOR 0.36, 95% CI 0.07–1.87; *p* = 0.222). Selected univariable and multivariable analyses are summarized in Table 5.

## 3. Discussion

In this 20-year cohort, *Achromobacter* bacteremia occurred predominantly as a late nosocomial event in patients with substantial prior healthcare and antimicrobial exposure. The principal findings were an association between prior carbapenem exposure and meropenem non-susceptibility, high 30-day mortality closely associated with host vulnerability and acute illness severity, attenuation of the crude association between active empiric therapy and mortality after adjustment for vasopressor use, and incomplete agreement between historical disk-diffusion and contemporary MIC interpretations.

The 50% 30-day mortality in our cohort exceeded that reported in most contemporary *Achromobacter* bloodstream infection series, including 10.2% by Bayless et al. and 23.1% by Pérez Barragán et al. [2,3], although earlier studies reported mortality up to 47.5% using heterogeneous outcome definitions [7,8]. This difference may reflect our tertiary referral population, which included patients with substantial comorbidity, antimicrobial exposure, and acute illness severity, limiting direct comparisons across studies (Table 6).

Contemporary data on uncommon non-fermenting Gram-negative bloodstream infections similarly highlight the interaction between healthcare exposure, diagnostic complexity, and antimicrobial resistance. Casale et al. reported frequent prolonged healthcare exposure and intravascular-device use, with *Achromobacter* spp. showing complex resistance phenotypes [11]. However, mortality varied substantially across organisms and clinical settings, reinforcing that organism identification and resistance phenotype should be interpreted together with acute illness severity and host factors.

In our cohort, higher NEWS and vasopressor requirement were associated with 30-day mortality, and vasopressor use remained strongly associated with mortality after adjustment, consistent with previous *Achromobacter* cohorts [9,10]. Shorter treatment duration among non-survivors likely reflected early death or transition to palliative care rather than harm from shorter therapy and is therefore subject to reverse causation and survivorship bias.

Antecedent antimicrobial exposure was common across published bacteremia cohorts [2,3,9]. Prior carbapenem exposure was associated with substantially higher odds of meropenem non-susceptibility in our cohort. Direct clinical evidence establishing antecedent carbapenem exposure as a patient-level risk factor for meropenem resistance in *Achromobacter* remains limited. Nevertheless, acquired meropenem resistance has been documented to emerge during therapy in sequential clinical isolates. Fleurbaaij et al. demonstrated that meropenem-resistant and susceptible *A. xylosoxidans* isolates obtained sequentially from the same patient were clonally related, supporting within-host emergence of resistance during treatment; the resistant isolate showed increased production of the AXC β-lactamase, which demonstrated carbapenem-hydrolyzing activity [12]. Experimental evidence also supports an efflux-mediated mechanism: Magallon et al. showed that overexpression of the AxyABM RND efflux system increased meropenem resistance, while inactivation of *axyB* reduced the meropenem MIC by 64-fold in an experimentally selected resistant mutant [13]. Genomic studies have additionally described intrinsic and acquired resistance determinants, including RND-type efflux systems and acquired carbapenemases, in *A. xylosoxidans* [14]. However, our observational data cannot establish that prior carbapenem exposure directly selected for meropenem resistance, and prior carbapenem use may also reflect greater cumulative healthcare and antimicrobial exposure. Molecular characterization of resistant isolates was not available in this study. Breakthrough bacteremia during ongoing systemic therapy, including therapy with an agent subsequently reported as susceptible, further suggests that in vitro activity may be insufficient in the presence of severe host illness, high organism burden, biofilm-associated infection, inadequate drug exposure, or an uncontrolled source.

Infection-source distribution differed from previous cohorts, in which catheter-related infection or central venous catheter (CVC) exposure was more prominent [2,3,7,8]. Primary bacteremia predominated in our cohort, whereas CRBSI accounted for fewer than one-fifth of cases. At our institution, suspected CRBSI has generally been evaluated using paired blood cultures and differential time to positivity, while catheter-tip or quantitative catheter cultures are not routinely performed. Because differential time to positivity (DTP) has imperfect sensitivity, some episodes classified as primary bacteremia may have represented catheter-associated infections without microbiological confirmation.

A noteworthy infection source was prosthetic aortic valve endocarditis in a 65-year-old woman four months after bioprosthetic valve replacement. Persistent fever and *Achromobacter*-positive blood cultures were accompanied by echocardiographic and cardiac computed tomography (CT) findings concerning for prosthetic valve endocarditis with perivalvular involvement. She initially received eight weeks of intravenous ceftazidime, but persistent imaging abnormalities ultimately prompted redo valve replacement; culture of the explanted valve again yielded *Achromobacter*. She subsequently received six weeks of oral ciprofloxacin and remained alive at follow-up. Although *Achromobacter* endocarditis is rare, previous prosthetic-valve cases have similarly required prolonged antimicrobial therapy combined with surgical source control [15,16,17].

Our historical AST profile differed from the recent multicenter experience reported by Bayless et al., in which bloodstream isolates were >90% susceptible to meropenem, piperacillin-tazobactam, and trimethoprim-sulfamethoxazole (TMP-SMX) [2]. The lower carbapenem and TMP-SMX susceptibility and poor ciprofloxacin activity in our cohort may reflect differences in patient selection, prior antimicrobial pressure, local resistance epidemiology, and testing methodology. In the small matched subset, discordance was concentrated in piperacillin-tazobactam and ceftazidime. Because historical disk results were interpreted mainly using *P. aeruginosa* surrogate breakpoints, whereas MICs were reinterpreted using contemporary CLSI Other Non-Enterobacterales criteria, discordance may reflect both methodological and breakpoint differences rather than disk-diffusion error alone. Changes in selected assignments under alternative CLSI and EUCAST frameworks further demonstrate that apparent susceptibility is partly breakpoint-dependent [5,6,18,19].

From a clinical laboratory perspective, this discordance has practical implications. Historical disk-diffusion results interpreted with surrogate breakpoints may overestimate activity and could provide false reassurance when piperacillin-tazobactam or ceftazidime is selected for a critically ill patient. Although our paired sample was small and the observed discrepancies reflect both methodological and breakpoint differences, susceptibility results derived from historical or non-organism-specific disk criteria should therefore be interpreted cautiously. Where available, contemporary MIC-based testing using an appropriate breakpoint framework should be prioritized for confirmation of clinically important isolates, particularly when treatment depends on a single β-lactam agent.

The crude association between active empiric therapy and lower mortality was substantially attenuated after adjustment for vasopressor use, highlighting confounding by acute illness severity. The adjusted estimate remained imprecise because of the limited number of deaths, and residual confounding by host factors, source, source control, and severity remains possible. Moreover, treatment timing was not modeled as a time-dependent exposure; patients with rapidly progressive shock may die before therapy can be optimized, whereas survivors have greater opportunity to receive active treatment. Larger time-aware studies are therefore needed. Previous small cohorts did not demonstrate a clear relationship between appropriate empiric therapy and mortality [3,10]. In our cohort, empiric regimens were frequently combinations, treatment selection was not randomized, and activity was defined using contemporaneous historical AST rather than a uniform modern standard. These findings suggest that early active therapy may be clinically important, although our observational data do not establish the comparative effectiveness of any individual antibiotic.

*Achromobacter* should also be considered in late-onset nosocomial bacteremia when blood cultures show Gram-negative bacilli but rapid multiplex testing does not identify an organism. *Achromobacter* is not included in the BIOFIRE Blood Culture Identification 2 (BCID2) Panel [20], and species-level identification within the genus may be challenging. Other clinically relevant off-panel non-fermenting Gram-negative bacilli include *Burkholderia* spp. and non-*aeruginosa Pseudomonas* spp., while less common organisms such as *Sphingomonas*, *Chryseobacterium*, *Myroides*, and *Acinetobacter* species outside the *A. calcoaceticus–baumannii* complex have also been reported [20,21]. Therefore, a “no target detected” BCID2 result from a positive blood culture does not exclude bacteremia and should prompt continued culture-based identification, including matrix-assisted laser desorption/ionization time-of-flight mass spectrometry (MALDI-TOF MS) and molecular methods when needed.

Based on these findings, piperacillin–tazobactam appears to be the most defensible local empiric backbone when *Achromobacter* bacteremia is suspected, with ceftazidime as a potential alternative. Both agents had high historically reported susceptibility in our cohort; however, historical disk testing appeared to overestimate their activity in the small paired-MIC subset, emphasizing the need for prompt MIC-based confirmation. Meropenem should not be relied upon as empiric monotherapy in patients with recent carbapenem exposure, among whom meropenem non-susceptibility reached 50%, whereas TMP-SMX and ciprofloxacin provided insufficient local coverage for routine empiric monotherapy. Empiric selection should therefore incorporate recent antimicrobial exposure, previous patient-specific isolates, and the local antibiogram, followed by prompt susceptibility-guided modification and source control. Our data do not demonstrate a benefit of routine combination therapy.

For multidrug-resistant isolates, therapeutic options become substantially more limited. Contemporary surveillance of minor non-fermenting Gram-negative bacilli has likewise demonstrated substantial resistance among *A. xylosoxidans* isolates and marked dependence of susceptibility estimates on the testing methodology [22]. Carbapenem resistance is particularly challenging because *Achromobacter* may combine intrinsic mechanisms, including multidrug efflux, with acquired metallo-β-lactamases (MBLs), including IMP-, VIM-, and more recently NDM-type enzymes [1,23]. Because avibactam, relebactam, and vaborbactam do not inhibit class B MBLs, ceftazidime-avibactam, imipenem-relebactam, and meropenem-vaborbactam cannot be assumed to restore activity when an MBL is the dominant resistance mechanism [24].

For MBL-producing Enterobacterales, aztreonam-avibactam or ceftazidime-avibactam combined with aztreonam provides a mechanism-directed strategy by pairing MBL-stable aztreonam with inhibition of accompanying serine β-lactamases. However, this paradigm cannot be directly extrapolated to *Achromobacter*, because aztreonam has intrinsically poor activity against this genus, in part owing to extrusion by the AxyABM efflux system [25]. Cefiderocol has demonstrated the most consistent activity, with an MIC50/MIC90 of 0.06/0.5 mg/L in a multinational collection of 334 *Achromobacter* isolates, including retained activity against many carbapenem-non-susceptible isolates; however, most isolates were respiratory and clinical experience remains limited [4,26]. Among non-β-lactams, minocycline and sitafloxacin have shown favorable in vitro activity, whereas tigecycline activity is inconsistent and its pharmacokinetic profile limits its suitability as monotherapy for bloodstream infection; eravacycline has shown low MICs but lacks validated breakpoints and clinical outcome data [27,28,29,30]. These agents should therefore not alter empiric treatment recommendations and should be reserved for individualized, MIC-guided salvage therapy when better-supported active agents are unavailable.

Although 44.9% of eligible episodes occurred during 2023–2025, a true increase in incidence cannot be inferred because annual denominators were unavailable and organism identification changed from phenotypic methods to MALDI-TOF during the study period. In addition, the study was not designed to evaluate hospital outbreaks or infection-control interventions, and molecular typing was unavailable to assess clonality among isolates from 2023 to 2025. Accordingly, the apparent temporal clustering may reflect changes in diagnostic ascertainment, patient case mix, antimicrobial pressure, or true transmission. Denominator-based surveillance incorporating microbiology testing volume and molecular epidemiology would be needed to distinguish among these possibilities.

This study has several strengths. It provides a detailed characterization of a rare bloodstream infection over a 20-year period, incorporating patient-level clinical severity, antecedent antimicrobial exposure, treatment, microbiological findings, and mortality. Restriction of the primary analysis to the first eligible episode per patient minimized within-patient clustering, while the availability of matched disk-diffusion and MIC results in a subset allowed exploration of the effect of testing methods and breakpoint frameworks on susceptibility interpretation.

Several limitations should be acknowledged. First, this was a retrospective, single-center study conducted at a tertiary-care university referral center serving Southern Thailand. The hospital likely receives a disproportionate number of patients with complex underlying diseases, extensive prior healthcare and antimicrobial exposure, and severe acute illness. This referral bias may have contributed to the high observed mortality and limits the generalizability of our findings to community hospitals and less severely ill populations. Second, the small cohort and 23 mortality events limited the number of variables that could be included in multivariable models, and residual confounding by unmeasured host, treatment, and source-control factors remains possible. Missing data were present for selected laboratory variables, particularly lactate (12/47) and bilirubin (6/47), although most clinical variables and the primary outcome were nearly complete. Historical organism-identification and AST methods evolved during the 20-year study period, species-level identification was not uniformly reliable, and raw disk-zone diameters were not consistently available for retrospective reinterpretation. Finally, the automated MIC analysis included only a small available subset, and differences between historical disk-diffusion practices and contemporary MIC breakpoint frameworks limit conclusions regarding categorical agreement.

Future work should validate the apparent relationship between prior carbapenem exposure and resistance in larger multicenter cohorts, apply contemporary *Achromobacter*-specific AST criteria to reference MIC data, and evaluate early effective therapy using time-aware methods.

## 4. Materials and Methods

### 4.1. Study Design and Setting

We conducted a retrospective cohort study at Songklanagarind Hospital, a tertiary-care university hospital in Southern Thailand. Adults aged ≥18 years with at least one blood culture yielding *Achromobacter* spp. between 1 January 2006 and 31 December 2025 were screened. The first eligible bacteremia episode per patient was used for the primary clinical and mortality analyses, while all eligible episodes were retained for temporal analyses. Because all eligible cases during the predefined study period were included, no a priori sample-size calculation was performed. Records yielding organisms other than *Achromobacter* spp., including *Alcaligenes faecalis*, and non-blood specimens inoculated into blood-culture bottles were excluded. The historical designation *Alcaligenes xylosoxidans* was considered eligible as a former designation of *Achromobacter xylosoxidans*.

### 4.2. Episode Definition and Index Time

The index time (T0) was the collection date and time of the first positive blood culture in the eligible episode. Repeat *Achromobacter*-positive blood cultures within 14 days were considered part of the same episode. A subsequent positive culture more than 14 days later was considered a new episode when the previous infection had clinically improved and/or blood cultures had cleared.

### 4.3. Clinical Variables and Antimicrobial Exposures

Clinical data were abstracted from electronic medical records, and microbiological data from the clinical microbiology laboratory database. Variables included demographics, healthcare exposures, comorbidities and immunosuppressive exposures, devices at T0, infection source, source control, antimicrobial therapy, and outcomes. Comorbidities were grouped into prespecified, non-mutually exclusive categories shown in Table 1. Chronic cardiac disease included ischemic heart disease/coronary artery disease, clinically significant valvular heart disease or prior valve surgery, cardiomyopathy, and atrial fibrillation. Chronic lung disease included chronic obstructive pulmonary disease and chronic interstitial lung disease. Nosocomial onset was defined as T0 > 48 h after admission; non-nosocomial episodes were classified as healthcare-associated when relevant healthcare exposure had occurred within 90 days. Infection sources were categorized as primary bacteremia, CRBSI, VAP with septicemia, UTI, prosthetic valve endocarditis, or other/mixed sources based on available clinical and microbiological data. Prior systemic antimicrobial exposure was defined as ≥1 dose during the 90 days before T0 for an indication preceding the index infection; carbapenem and colistin exposures were recorded separately. Devices were considered present at T0 only if present at the index blood-culture time. Ongoing antimicrobial therapy was defined as systemic therapy being administered at T0, and we recorded whether ≥1 ongoing agent was subsequently reported as susceptible by contemporaneous AST.

### 4.4. Physiologic and Laboratory Data

Vital signs and laboratory values closest to T0 were extracted. NEWS was calculated from its standard components using measurements recorded closest to T0. Laboratory variables included white blood cell and absolute neutrophil counts, hemoglobin, platelet count, creatinine, total and direct bilirubin, albumin, and lactate.

### 4.5. Microbiological Identification and AST

Historical organism identification methods changed during the study period. Before implementation of MALDI-TOF in approximately 2017, non-fermenting Gram-negative bacilli were identified using phenotypic/biochemical methods with flagellar staining; MALDI-TOF was subsequently used. Because historical species-level discrimination was imperfect and reporting practices changed, the primary analysis was performed at the *Achromobacter* genus level, while original organism labels were retained. No polymerase chain reaction (PCR), whole-genome sequencing, or other molecular testing for carbapenemase genes, efflux-system determinants, or other antimicrobial-resistance genes was performed on the study isolates.

Historical susceptibility categories susceptible/intermediate/resistant (S/I/R) were retained as originally reported because they represent the information available to treating clinicians at the time. For the primary historical susceptibility analysis, the first blood isolate from the first eligible bacteremia episode of each patient was used. According to the clinical microbiology laboratory’s historical practice, disk-diffusion results for *Achromobacter* spp. were interpreted using CLSI *P. aeruginosa* surrogate breakpoints for most antimicrobial agents; trimethoprim-sulfamethoxazole was interpreted using the laboratory’s Other Non-Enterobacterales criteria. Raw historical zone diameters were not consistently available and therefore could not be retrospectively reinterpreted. Automated MIC testing was performed using the Sensititre™ system (Thermo Fisher Scientific, Waltham, MA, USA), a commercial broth microdilution platform, according to the manufacturer’s instructions. In the automated MIC subset, only panels linked to blood isolates were included; confirmed MIC panels from non-blood specimens were excluded. Exact source-lab-number matches, together with one laboratory-confirmed pair originating from different blood-culture bottles collected simultaneously, were used for the primary paired disk-MIC comparison. Numeric MICs were retrospectively categorized using CLSI M100, 36th edition (2026), Other Non-Enterobacterales [18]. CLSI notes that disk diffusion has not been systematically studied and is not recommended for this organism group; therefore, the Other Non-Enterobacterales criteria were applied only to numeric MICs, not to historical disk zones. Colistin was not assigned a contemporary S/I/R category for the primary agreement analysis. Sensitivity analyses reclassified MICs using current CLSI *P. aeruginosa* breakpoints and, for agents with available organism-specific criteria, EUCAST v16.1 *A. xylosoxidans* breakpoints [19].

### 4.6. Treatment Definitions and Outcomes

Empiric therapy was defined as systemic antimicrobial therapy for the index infection administered before definitive organism identification and susceptibility results, whether initiated before or after T0. Therapy was considered active if ≥1 administered agent was subsequently reported susceptible by contemporaneous AST; therapy initiated after susceptibility results became available was classified as directed therapy. The primary outcome was 30-day all-cause mortality. Secondary outcomes included microbiological clearance or persistence, treatment duration, and recurrent bacteremia. Persistent bacteremia was defined as recovery of *Achromobacter* spp. from the first follow-up blood culture after initiation of empiric therapy. Follow-up cultures were obtained at the treating physician’s discretion, generally within 2–3 days; patients without follow-up cultures were excluded from persistence analyses.

### 4.7. Statistical Analysis

Analyses were performed using R Statistical Software (R Foundation for Statistical Computing, Vienna, Austria). Continuous variables were summarized as medians (IQR) and compared using the Mann–Whitney U test; categorical variables were summarized as *n*/*N* (%) and compared using Fisher’s exact test. For selected binary mortality predictors, ORs and 95% CIs were estimated using conditional exact methods, with two-sided *p* values from Fisher’s exact test. Continuous predictors were evaluated using univariable logistic regression with Wald 95% CIs. Given only 23 deaths, multivariable modeling was deliberately parsimonious. The prespecified model included active empiric therapy as the exposure of interest and vasopressor use at T0 as the principal severity covariate; other physiologic variables were excluded to limit overfitting and collinearity. Missing data were handled using available-case analysis for descriptive comparisons and complete-case analysis for regression models, without imputation of the primary outcome. Analyses were exploratory, *p* values were not adjusted for multiple comparisons, and statistical significance was defined as two-sided *p* < 0.05.

### 4.8. Ethics

The study was conducted in accordance with the Declaration of Helsinki and was approved by the Human Research Ethics Committee, Faculty of Medicine, Prince of Songkla University (REC.69-418-14-1). Because of the retrospective design, the requirement for individual informed consent was waived.

## 5. Conclusions

*Achromobacter* bacteremia in this tertiary-care cohort occurred predominantly as a nosocomial infection in heavily antibiotic-exposed patients and carried high 30-day mortality. Prior carbapenem exposure was associated with meropenem non-susceptibility, while acute physiologic severity was strongly associated with death. Active empiric therapy was associated with lower crude mortality, but this association attenuated after adjustment for vasopressor use. In a small matched MIC subset, historical disk and contemporary MIC interpretations were not fully concordant.

## Figures and Tables

**Figure 1:**
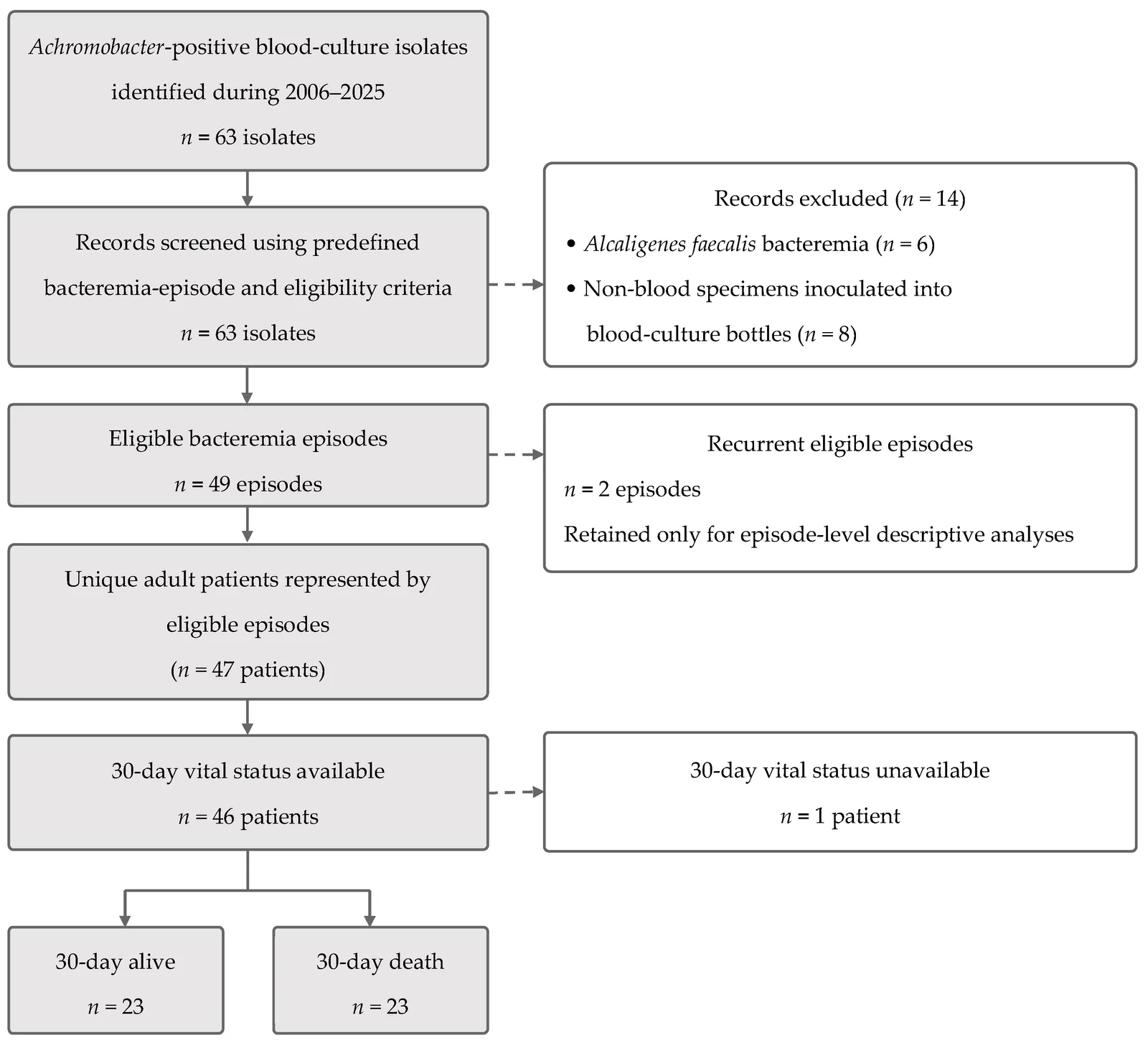
Study flow diagram.

**Figure 2:**
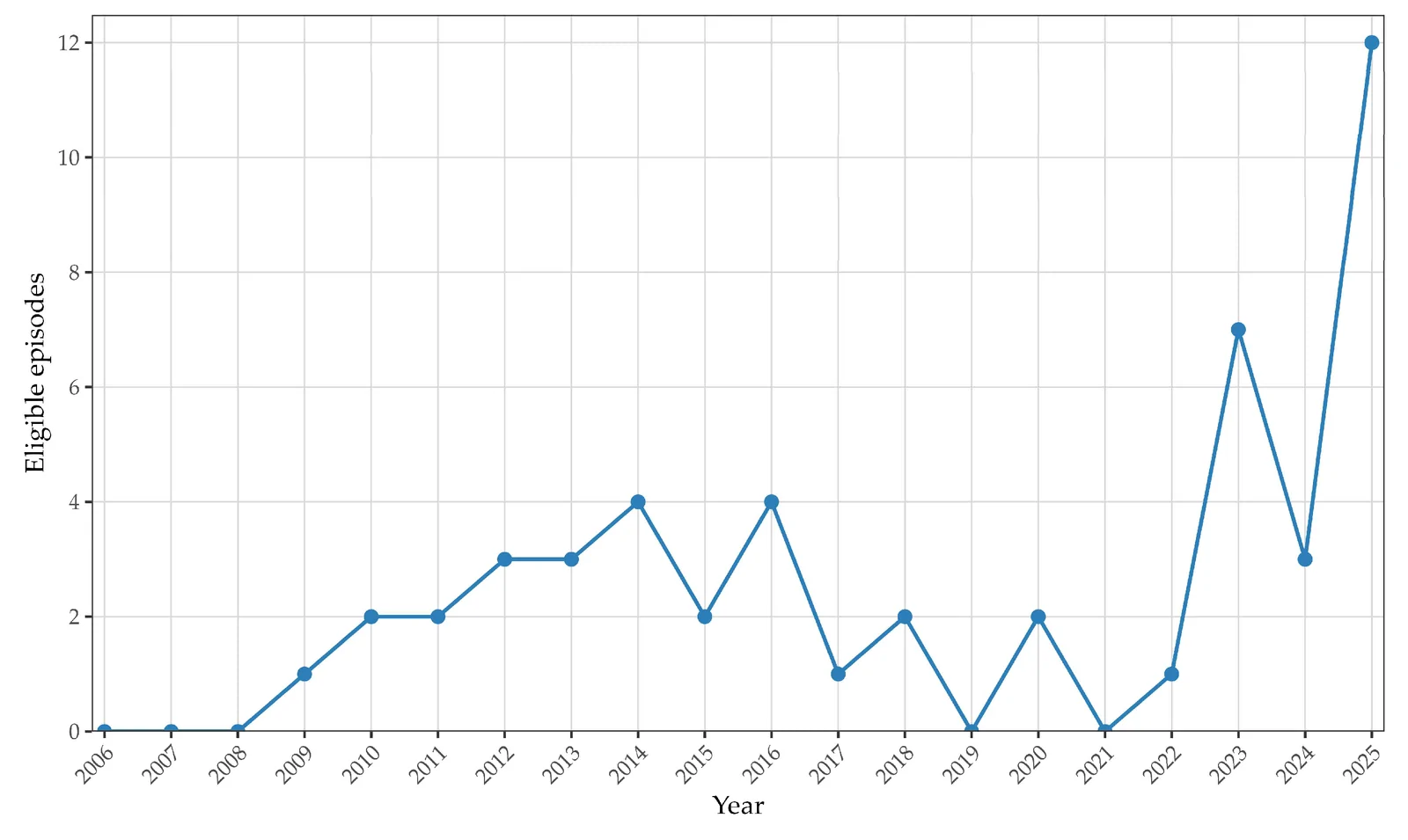
Annual number of eligible *Achromobacter* bacteremia episodes during 2006–2025.

**Table 1:** Baseline clinical and laboratory characteristics according to 30-day outcome.

Characteristic	Overall	30-d Death	30-d Alive	*p* Value
Demographic and underlying conditions				
Age, years	60.0 (51.5–71.0)	60.0 (49.5–71.5)	63.0 (55.5–70.5)	0.717
Male sex	33/47 (70.2%)	17/23 (73.9%)	15/23 (65.2%)	0.749
Any malignancy	22/47 (46.8%)	15/23 (65.2%)	6/23 (26.1%)	0.017
Solid malignancy	17/47 (36.2%)	11/23 (47.8%)	5/23 (21.7%)	0.120
Hematologic malignancy	5/47 (10.6%)	4/23 (17.4%)	1/23 (4.3%)	0.346
Hypertension	11/47 (23.4%)	4/23 (17.4%)	7/23 (30.4%)	0.491
Diabetes mellitus	6/47 (12.8%)	2/23 (8.7%)	4/23 (17.4%)	0.665
Chronic kidney disease/ESRD	6/47 (12.8%)	1/23 (4.3%)	5/23 (21.7%)	0.187
Chronic cardiac disease	8/47 (17.0%)	3/23 (13.0%)	5/23 (21.7%)	0.699
Cerebrovascular disease	5/47 (10.6%)	2/23 (8.7%)	3/23 (13.0%)	1.000
Chronic lung disease	2/47 (4.3%)	2/23 (8.7%)	0/23 (0.0%)	0.489
Chronic liver disease	2/47 (4.3%)	0/23 (0.0%)	2/23 (8.7%)	0.489
Autoimmune/inflammatory disease	3/47 (6.4%)	1/23 (4.3%)	2/23 (8.7%)	1.000
HIV infection	1/47 (2.1%)	0/23 (0.0%)	1/23 (4.3%)	1.000
Healthcare and antimicrobial exposures				
Days from admission to T0	15.0 (5.0–49.5)	26.0 (9.5–56.5)	12.0 (5.0–43.0)	0.350
Nosocomial onset	43/47 (91.5%)	21/23 (91.3%)	21/23 (91.3%)	1.000
ICU exposure within 90 d	23/47 (48.9%)	14/23 (60.9%)	9/23 (39.1%)	0.238
Prior hospitalization within 90 d	26/47 (55.3%)	15/23 (65.2%)	11/23 (47.8%)	0.373
Prior systemic antibiotic within 90 d	43/45 (95.6%)	21/23 (91.3%)	21/21 (100.0%)	0.489
Prior carbapenem within 90 d	26/45 (57.8%)	17/23 (73.9%)	8/21 (38.1%)	0.032
Prior colistin within 90 d	17/45 (37.8%)	10/23 (43.5%)	7/21 (33.3%)	0.548
Chemotherapy/immunosuppression within 90 d	9/47 (19.1%)	7/23 (30.4%)	2/23 (8.7%)	0.135
TPN within 90 d	10/47 (21.3%)	8/23 (34.8%)	2/23 (8.7%)	0.071
Corticosteroid within 90 d	17/47 (36.2%)	12/23 (52.2%)	5/23 (21.7%)	0.065
Devices and organ support at T0				
CVC present at T0	17/47 (36.2%)	10/23 (43.5%)	7/23 (30.4%)	0.542
Arterial line at T0	16/47 (34.0%)	12/23 (52.2%)	4/23 (17.4%)	0.029
Mechanical ventilation at T0	27/47 (57.4%)	16/23 (69.6%)	11/23 (47.8%)	0.231
Vasopressor at T0	15/47 (31.9%)	13/23 (56.5%)	2/23 (8.7%)	0.001
RRT at T0	8/47 (17.0%)	4/23 (17.4%)	4/23 (17.4%)	1.000
Physiologic variables at T0				
Temperature, °C	38.0 (37.4–38.8)	37.9 (37.0–38.6)	38.3 (37.7–39.0)	0.153
SBP, mmHg	120 (104–139)	109 (92–117.5)	124 (116.5–146)	0.002
MAP, mmHg	83.7 (74.3–97.0)	80.7 (66.5–87.7)	89.0 (81.7–102.9)	0.008
Heart rate, /min	111 (89.5–123)	112 (95.5–125.5)	106 (87.5–117.5)	0.272
Respiratory rate, /min	24 (20–27.5)	27 (22.5–31)	22 (19.5–24)	0.006
NEWS at T0	5.0 (2.0–7.0)	6.0 (3.0–8.0)	3.0 (1.0–5.0)	0.005
Laboratory variables closest to T0				
WBC, cells/mm^3^	12,520 (6710–17,040)	14,520 (6535–22,780)	12,120 (7275–13,550)	0.099
ANC, cells/mm^3^	10,873 (5721–14,967)	12,676 (5366–21,206)	9924 (6029–11,698)	0.132
Hemoglobin, g/dL	9.2 (8.3–10.0)	9.1 (8.3–10.0)	9.4 (8.5–10.0)	0.575
Platelet, ×10^3^/µL	167.0 (77.0–287.5)	81.0 (39.5–207.0)	271.0 (136.0–305.0)	0.006
Creatinine, mg/dL	1.21 (0.68–2.28)	1.45 (0.78–2.28)	0.93 (0.56–2.05)	0.087
Total bilirubin, mg/dL	1.02 (0.58–3.70)	1.85 (0.83–4.00)	0.68 (0.42–1.44)	0.035
Direct bilirubin, mg/dL	0.55 (0.25–3.00)	1.34 (0.48–3.25)	0.28 (0.21–0.49)	0.008
Albumin, g/dL	2.50 (2.23–2.90)	2.40 (1.80–2.60)	2.85 (2.40–3.48)	0.001
Lactate, mmol/L	2.10 (1.15–3.15)	2.40 (1.88–3.47)	1.10 (0.85–2.03)	0.009

Values are presented as median (IQR) or *n*/*N* (%). One patient with unavailable 30-day vital status was included only in the overall column. Abbreviations: ANC, absolute neutrophil count; CVC, central venous catheter; ESRD, end-stage renal disease; HIV, human immunodeficiency virus; ICU, intensive care unit; IQR, interquartile range; MAP, mean arterial pressure; NEWS, National Early Warning Score; RRT, renal replacement therapy; SBP, systolic blood pressure; T0, index blood-culture collection time; TPN, total parenteral nutrition; WBC, white blood cell count.

**Table 2:** Infection characteristics, treatment, and outcomes.

Variable	Overall	30-d Death	30-d Alive	*p* Value
Source of bacteremia				0.199
Primary bacteremia	26/47 (55.3%)	11/23 (47.8%)	14/23 (60.9%)	—
CRBSI	9/47 (19.1%)	6/23 (26.1%)	3/23 (13.0%)	—
VAP	4/47 (8.5%)	3/23 (13.0%)	1/23 (4.3%)	—
UTI	3/47 (6.4%)	0/23 (0.0%)	3/23 (13.0%)	—
Prosthetic valve endocarditis	1/47 (2.1%)	0/23 (0.0%)	1/23 (4.3%)	—
Other/mixed source	4/47 (8.5%)	3/23 (13.0%)	1/23 (4.3%)	—
Polymicrobial bacteremia	13/47 (27.7%)	8/23 (34.8%)	5/23 (21.7%)	0.514
Antimicrobial exposure and treatment				
Ongoing systemic antimicrobial therapy at T0	23/47 (48.9%)	15/23 (65.2%)	7/23 (30.4%)	0.038
Carbapenem and/or colistin at T0 among patients receiving ongoing therapy	14/23 (60.9%)	11/15 (73.3%)	3/7 (42.9%)	0.343
Receiving ≥1 ongoing agent subsequently reported susceptible by contemporaneous AST	7/23 (30.4%)	5/15 (33.3%)	2/7 (28.6%)	1.000
Active empiric therapy	34/47 (72.3%)	13/23 (56.5%)	20/23 (87.0%)	0.047
Directed combination therapy	5/47 (10.6%)	4/23 (17.4%)	1/23 (4.3%)	0.346
Treatment duration, days	10 (5–14)	5 (3–9)	14 (12–14)	<0.001
Early clinical course				
Hospital length of stay, days	44 (16–76.5)	41 (14–62)	52 (25.5–93)	0.170
Death within 7 days of T0	11/47 (23.4%)	11/23 (47.8%)	0/23 (0.0%)	<0.001
Microbiological follow-up				
Follow-up blood culture obtained	34/47 (72.3%)	12/23 (52.2%)	21/23 (91.3%)	0.007
Persistent *Achromobacter* bacteremia among patients with follow-up cultures	8/34 (23.5%)	6/12 (50.0%)	2/21 (9.5%)	0.015

Values are presented as median (IQR) or *n*/*N* (%). One patient with unavailable 30-day vital status was included only in the overall column. The symbol “—” indicates that individual source categories were not tested separately. Abbreviations: AST, antimicrobial susceptibility testing; CRBSI, catheter-related bloodstream infection; IQR, interquartile range; T0, index blood-culture collection time; UTI, urinary tract infection; VAP, ventilator-associated pneumonia.

**Table 3:** Historical laboratory-reported antimicrobial susceptibility profile.

Agent	Tested, *n*	S, *n* (%)	I, *n* (%)	R, *n* (%)
Piperacillin-tazobactam	45	44 (97.8)	0 (0.0)	1 (2.2)
Ceftazidime	46	43 (93.5)	1 (2.2)	2 (4.3)
TMP-SMX	47	32 (68.1)	0 (0.0)	15 (31.9)
Imipenem	45	31 (68.9)	1 (2.2)	13 (28.9)
Meropenem	46	31 (67.4)	2 (4.3)	13 (28.3)
Ciprofloxacin	47	7 (14.9)	6 (12.8)	34 (72.3)
Colistin	30	18 (60.0)	0 (0.0)	12 (40.0)

Historical susceptibility categories were retained as originally reported by the clinical microbiology laboratory; colistin results were not reinterpreted using contemporary breakpoints. Abbreviations: I, intermediate; R, resistant; S, susceptible; TMP-SMX, trimethoprim-sulfamethoxazole.

**Table 4:** Historical disk versus CLSI M100 2026 MIC categorical agreement among laboratory-confirmed paired blood isolates.

Drug	Comparable	Concordant	Discordant	Agreement	Disk S → MIC I/R
PTZ	6	4	2	66.7%	2
CAZ	7	4	3	57.1%	3
IPM	7	6	1	85.7%	0
MEM	7	7	0	100.0%	0
CIP	7	6	1	85.7%	0
TMP-SMX	7	6	1	85.7%	1

Agreement was calculated among paired isolates with comparable categorical results. “Disk S → MIC I/R” indicates historical disk-susceptible isolates categorized as intermediate or resistant using contemporary MIC criteria. Abbreviations: CAZ, ceftazidime; CIP, ciprofloxacin; CLSI, Clinical and Laboratory Standards Institute; I, intermediate; IPM, imipenem; MEM, meropenem; MIC, minimum inhibitory concentration; PTZ, piperacillin-tazobactam; R, resistant; S, susceptible; TMP-SMX, trimethoprim-sulfamethoxazole.

**Table 5:** Selected univariable associations and adjusted logistic regression analyses for 30-day mortality.

Model/Predictor	*n*	Effect Estimate (95% CI)	*p* Value
Univariable analyses			
Prior carbapenem within 90 d	44	4.43 (1.09–20.31)	0.032
Ongoing systemic antimicrobial therapy at T0	46	4.14 (1.07–17.73)	0.038
TPN within 90 d	46	5.40 (0.90–59.27)	0.071
Arterial line at T0	46	4.99 (1.15–26.69)	0.029
Vasopressor at T0	46	12.81 (2.27–138.37)	0.001
Corticosteroid within 90 d	46	3.81 (0.93–17.87)	0.065
Active empiric therapy	46	0.20 (0.03–0.98)	0.047
NEWS at T0, per point	46	1.39 (1.09–1.76)	0.007
SBP at T0, per 10 mmHg	46	0.72 (0.55–0.94)	0.016
Albumin, per 1 g/dL	45	0.14 (0.04–0.53)	0.004
Any malignancy	46	5.10 (1.28–23.00)	0.017
Parsimonious adjusted model: active empiric therapy + vasopressor use at T0			
Active empiric therapy	46	0.36 (0.07–1.87)	0.222
Vasopressor at T0	46	10.43 (1.88–57.93)	0.007

Binary predictors were analyzed using exact methods, whereas continuous predictors and adjusted models were analyzed using logistic regression. Reported *p*-values were not adjusted for multiple comparisons. Effect estimates are odds ratios (ORs) for univariable analyses and adjusted odds ratios (aORs) for the multivariable model. Abbreviations: aOR, adjusted odds ratio; CI, confidence interval; NEWS, National Early Warning Score; OR, odds ratio; SBP, systolic blood pressure; T0, index blood-culture collection time; TPN, total parenteral nutrition.

**Table 6:** Selected published cohorts of *Achromobacter* bacteremia compared with the present study.

Study	Setting and Population	Bacteremia Cohort	Reported Antimicrobial Exposure	Selected Clinical Characteristics	Mortality
Gómez-Cerezo et al., 2003 [7]	Single center, Spain; 10-year retrospective series	54 cases	NR	52/54 nosocomial; neoplasm was the most common underlying condition; catheter-related source in 35 (60%)	8/54 (15%) overall mortality
Aisenberg et al., 2004 [9]	MD Anderson Cancer Center, USA; patients with malignancy, 1989–2003	52 episodes in 46 patients	18/46 (39%) receiving antimicrobials at first positive blood culture	Hematologic malignancy 67%; neutropenia 52%; polymicrobial BSI 52%; sepsis syndrome and high APACHE II associated with mortality	7/46 (15%) infection-attributable mortality
Shie et al., 2005 [8]	Single medical center, northern Taiwan; 6-year series	40 cases	NR	Malignancy 57.5%; CVC 55%; neutropenia 30%; 72.5% healthcare-acquired	47.5% mortality attributed to bacteremia
Pérez Barragán et al., 2018 [3]	Single tertiary-care center, Spain; 2007–2017	13 episodes	7/13 (53.8%) within 30 days before bacteremia	12/13 hospital-acquired; CVC 46.1%	3/13 (23.1%) 30-day all-cause mortality
Bayless et al., 2025 [2]	Three Mayo Clinic tertiary-care centers, USA; 2013–2023	52 BSI cases	36/51 (70.6%) within 30 days before BSI	Immunocompromised 59.6%; CVC 73.1%; potential catheter-related source 57.7%; polymicrobial BSI 48.1%	5/49 (10.2%) 30-day all-cause mortality
Pekince et al., 2026 [10]	Critically ill ICU patients, Türkiye; retrospective cohort	26 patients	NR	All met Sepsis-3 criteria; non-survivors were older and had higher SOFA and APACHE II scores; all deaths occurred in mechanically ventilated patients with a presumed pulmonary source	7/26 (26.9%) overall mortality
Present study	Tertiary-care university hospital, Thailand; 2006–2025	49 episodes in 47 patients	43/45 (95.6%) within 90 days before T0; 23/47 (48.9%) receiving ongoing therapy at T0	Nosocomial 91.5%; vasopressor use at T0 31.9%; mechanical ventilation 57.4%	23/46 (50.0%) 30-day all-cause mortality

Antimicrobial-exposure definitions and assessment windows differed across studies; percentages are descriptive and should not be directly compared. Mortality definitions are presented as originally reported in each study. Abbreviations: APACHE II, Acute Physiology and Chronic Health Evaluation II; BSI, bloodstream infection; CVC, central venous catheter; ICU, intensive care unit; NR, not reported; SOFA, Sequential Organ Failure Assessment; T0, index blood-culture collection time.

## Data Availability

The datasets generated and/or analyzed during the current study are not publicly available because they contain information derived from patient medical records and are subject to institutional privacy and ethical restrictions. De-identified data may be available from the corresponding author upon reasonable request and subject to approval by the relevant institutional and ethics authorities.

## Supplementary Materials

The following supporting information can be downloaded at https://www.mdpi.com/article/10.3390/antibiotics15100953/s1: Table S1: De-identified automated MIC results and corresponding historical disk-diffusion susceptibility categories among laboratory-confirmed paired blood isolates; Table S2: Categorical agreement between historical disk-diffusion results and automated MIC interpretations under alternative breakpoint frameworks.

## Abbreviations

The following abbreviations are used in this manuscript:

Abbreviation	Definition
AST	Antimicrobial susceptibility testing
BSI	Bloodstream infection
CAZ	Ceftazidime
CLSI	Clinical and Laboratory Standards Institute
CRBSI	Catheter-related bloodstream infection
CVC	Central venous catheter
DD	Disk diffusion
IPM	Imipenem
MALDI-TOF	Matrix-assisted laser desorption/ionization time-of-flight mass spectrometry
MEM	Meropenem
MIC	Minimum inhibitory concentration
NEWS	National Early Warning Score
PTZ	Piperacillin-tazobactam
PVE	Prosthetic valve endocarditis
RRT	Renal replacement therapy
TMP-SMX	Trimethoprim-sulfamethoxazole
T0	Index blood-culture collection time
VAP	Ventilator-associated pneumonia
