# Supplementary material for: Clinical Characteristics, Antimicrobial Susceptibility, Treatment, and Outcomes of Achromobacter Bacteremia: A 20-Year Retrospective Cohort Study
Source: Antibiotics (Basel). 2026 Sep 25;15(10):953. doi: 10.3390/antibiotics15100953 (PMC13700674; doi:10.3390/antibiotics15100953)
Supplement: Supplementary file 1 [file antibiotics-15-00953-s001.zip › Table S1. De-identified automated MIC results and corresponding historical disk-diffusion susceptibility categories among laboratory-confirmed paired blood isolates..pdf]

Table S1. De-identified automated MIC results and corresponding historical disk-diffusion susceptibility categories among laboratory-confirmed paired blood isolates.

Isolate	Antimicrobial agent	Automated MIC (mg/L)	CLSI Other Non-Enterobacterales category	Historical disk category
**AX-B01**	Piperacillin-tazobactam	≤8	S	S
**AX-B01**	Ceftazidime	8	S	S
**AX-B01**	Imipenem	8	I	R
**AX-B01**	Meropenem	>8	R	R
**AX-B01**	Ciprofloxacin	>2	R	R
**AX-B01**	TMP-SMX	≤1	S	S
**AX-B01**	Colistin	2	NI	S
**AX-B01**	Levofloxacin	>8	R	NT
**AX-B02**	Piperacillin-tazobactam	16	S	S
**AX-B02**	Ceftazidime	16	I	S
**AX-B02**	Imipenem	>8	R	R
**AX-B02**	Meropenem	>16	R	R
**AX-B02**	Ciprofloxacin	4	R	R
**AX-B02**	TMP-SMX	>4	R	R
**AX-B02**	Colistin	1	NI	NT
**AX-B02**	Levofloxacin	4	I	NT
**AX-B03**	Piperacillin-tazobactam	64	I	NT
**AX-B03**	Ceftazidime	16	I	I
**AX-B03**	Imipenem	>8	R	R
**AX-B03**	Meropenem	>8	R	R
**AX-B03**	Ciprofloxacin	>2	R	R
**AX-B03**	TMP-SMX	≤1	S	S
**AX-B03**	Colistin	≤1	NI	S
**AX-B03**	Levofloxacin	>8	R	NT
**AX-B04**	Piperacillin-tazobactam	32	I	S
**AX-B04**	Ceftazidime	16	I	S
**AX-B04**	Imipenem	>8	R	R
**AX-B04**	Meropenem	>8	R	R
**AX-B04**	Ciprofloxacin	>2	R	R
**AX-B04**	TMP-SMX	>4	R	R
**AX-B04**	Colistin	≤1	NI	NT

**AX-B04**	Levofloxacin	4	I	NT
**AX-B05**	Piperacillin-tazobactam	≤8	S	S
**AX-B05**	Ceftazidime	≤4	S	S
**AX-B05**	Imipenem	≤0.5	S	S
**AX-B05**	Meropenem	≤0.5	S	S
**AX-B05**	Ciprofloxacin	2	I	I
**AX-B05**	TMP-SMX	≤1	S	S
**AX-B05**	Colistin	2	NI	NT
**AX-B05**	Levofloxacin	2	S	NT
**AX-B06**	Piperacillin-tazobactam	≤8	S	S
**AX-B06**	Ceftazidime	2	S	S
**AX-B06**	Imipenem	≤0.5	S	S
**AX-B06**	Meropenem	≤0.5	S	S
**AX-B06**	Ciprofloxacin	1	S	R
**AX-B06**	TMP-SMX	>4	R	R
**AX-B06**	Colistin	≤1	NI	NT
**AX-B06**	Levofloxacin	2	S	NT
**AX-B07**	Piperacillin-tazobactam	>64	R	S
**AX-B07**	Ceftazidime	16	I	S
**AX-B07**	Imipenem	>8	R	R
**AX-B07**	Meropenem	>8	R	R
**AX-B07**	Ciprofloxacin	>2	R	R
**AX-B07**	TMP-SMX	>4	R	S
**AX-B07**	Colistin	4	NI	R
**AX-B07**	Levofloxacin	>8	R	NT

Only laboratory-confirmed paired blood isolates were included. Isolate identifiers were newly assigned and cannot be linked to patient, laboratory, or collection identifiers. Historical disk-diffusion categories were retained as originally reported by the clinical microbiology laboratory. Automated MICs were retrospectively interpreted using CLSI M100, 36th edition, Other Non-Enterobacterales criteria. Colistin was not assigned a contemporary susceptibility category because no applicable susceptible breakpoint was available. Levofloxacin was not included in the categorical-agreement analysis because corresponding historical disk results were unavailable. Abbreviations: CLSI, Clinical and Laboratory Standards Institute; I, intermediate; MIC, minimum inhibitory concentration; NI, no applicable interpretation; NT, not tested or not reported; R, resistant; S, susceptible; TMP-SMX, trimethoprim-sulfamethoxazole.
