# Supplementary material for: Clinical Characteristics, Antimicrobial Susceptibility, Treatment, and Outcomes of Achromobacter Bacteremia: A 20-Year Retrospective Cohort Study
Source: Antibiotics (Basel). 2026 Sep 25;15(10):953. doi: 10.3390/antibiotics15100953 (PMC13700674; doi:10.3390/antibiotics15100953)
Supplement: Supplementary file 1 [file antibiotics-15-00953-s001.zip › Table S2. Categorical agreement between historical disk-diffusion results and automated MIC interpretations under alternative breakpoint frameworks..pdf]

Table S2. Categorical agreement between historical disk-diffusion results and automated MIC interpretations under alternative breakpoint frameworks.

Table S2A. Retrospective MIC categories by isolate and breakpoint framework

Isolate	Agent	Historical disk	MIC (mg/L)	CLSI Other Non-Enterobacterales	CLSI *P. aeruginosa*	EUCAST *A. xylosoxidans*
**AX-B01**	PTZ	S	≤8	S	S	Indeterminate
**AX-B01**	CAZ	S	8	S	S	NI
**AX-B01**	IPM	R	8	I	R	NI
**AX-B01**	MEM	R	>8	R	R	R
**AX-B01**	CIP	R	>2	R	R	NI
**AX-B01**	TMP-SMX	S	≤1	S	NI	Indeterminate
**AX-B02**	PTZ	S	16	S	S	R
**AX-B02**	CAZ	S	16	I	I	NI
**AX-B02**	IPM	R	>8	R	R	NI
**AX-B02**	MEM	R	>16	R	R	R
**AX-B02**	CIP	R	4	R	R	NI
**AX-B02**	TMP-SMX	R	>4	R	NI	R
**AX-B03**	PTZ	NT	64	I	R	R
**AX-B03**	CAZ	I	16	I	I	NI
**AX-B03**	IPM	R	>8	R	R	NI
**AX-B03**	MEM	R	>8	R	R	R
**AX-B03**	CIP	R	>2	R	R	NI
**AX-B03**	TMP-SMX	S	≤1	S	NI	Indeterminate
**AX-B04**	PTZ	S	32	I	I	R
**AX-B04**	CAZ	S	16	I	I	NI
**AX-B04**	IPM	R	>8	R	R	NI
**AX-B04**	MEM	R	>8	R	R	R
**AX-B04**	CIP	R	>2	R	R	NI
**AX-B04**	TMP-SMX	R	>4	R	NI	R
**AX-B05**	PTZ	S	≤8	S	S	Indeterminate
**AX-B05**	CAZ	S	≤4	S	S	NI
**AX-B05**	IPM	S	≤0.5	S	S	NI
**AX-B05**	MEM	S	≤0.5	S	S	S
**AX-B05**	CIP	I	2	I	R	NI
**AX-B05**	TMP-SMX	S	≤1	S	NI	Indeterminate
**AX-B06**	PTZ	S	≤8	S	S	Indeterminate
**AX-B06**	CAZ	S	2	S	S	NI

**AX-B06**	IPM	S	≤0.5	S	S	NI
**AX-B06**	MEM	S	≤0.5	S	S	S
**AX-B06**	CIP	R	1	S	I	NI
**AX-B06**	TMP-SMX	R	>4	R	NI	R
**AX-B07**	PTZ	S	>64	R	R	R
**AX-B07**	CAZ	S	16	I	I	NI
**AX-B07**	IPM	R	>8	R	R	NI
**AX-B07**	MEM	R	>8	R	R	R
**AX-B07**	CIP	R	>2	R	R	NI
**AX-B07**	TMP-SMX	S	>4	R	NI	R

Table S2B. Summary of categorical agreement by breakpoint framework

**Breakpoint framework**	**Agent**	**Comparable pairs, *n***	**Concordant, *n***	**Discordant, *n***	**Agreement, %**	**Historical disk S → MIC I/R, *n***
**CLSI Other Non-Enterobacterales**	PTZ	6	4	2	66.7	2
**CLSI Other Non-Enterobacterales**	CAZ	7	4	3	57.1	3
**CLSI Other Non-Enterobacterales**	IPM	7	6	1	85.7	0
**CLSI Other Non-Enterobacterales**	MEM	7	7	0	100.0	0
**CLSI Other Non-Enterobacterales**	CIP	7	6	1	85.7	0
**CLSI Other Non-Enterobacterales**	TMP-SMX	7	6	1	85.7	1
**CLSI *P. aeruginosa***	PTZ	6	4	2	66.7	2
**CLSI *P. aeruginosa***	CAZ	7	4	3	57.1	3
**CLSI *P. aeruginosa***	IPM	7	7	0	100.0	0
**CLSI *P. aeruginosa***	MEM	7	7	0	100.0	0
**CLSI *P. aeruginosa***	CIP	7	5	2	71.4	0
**CLSI *P. aeruginosa***	TMP-SMX	0	—	—	—	—
**EUCAST *A. xylosoxidans***	PTZ	3	0	3	0.0	3
**EUCAST *A. xylosoxidans***	CAZ	0	—	—	—	—
**EUCAST *A. xylosoxidans***	IPM	0	—	—	—	—

**EUCAST** ***A. xylosoxidans***	MEM	7	7	0	100.0	0
**EUCAST** ***A. xylosoxidans***	CIP	0	—	—	—	—
**EUCAST** ***A. xylosoxidans***	TMP-SMX	4	3	1	75.0	1

Agreement was assessed only when both a historical disk category and an unambiguous retrospective MIC category were available. Censored MIC values that crossed a breakpoint could not be categorized reliably and were classified as indeterminate. For example, a piperacillin-tazobactam MIC of ≤8 mg/L could not be categorized using the EUCAST *A. xylosoxidans* breakpoint of S ≤4 mg/L/R >4 mg/L. Similarly, TMP-SMX MICs reported as ≤1 mg/L could not be categorized using the EUCAST breakpoint because the reported dilution range crossed the organism-specific breakpoint. Indeterminate and NI results were excluded from agreement denominators. EUCAST v16.1 provides *A. xylosoxidans*-specific criteria for piperacillin-tazobactam, meropenem, and TMP-SMX but not for ceftazidime, imipenem, or ciprofloxacin. CLSI *P. aeruginosa* does not provide an applicable TMP-SMX breakpoint. Abbreviations: CAZ, ceftazidime; CIP, ciprofloxacin; CLSI, Clinical and Laboratory Standards Institute; EUCAST, European Committee on Antimicrobial Susceptibility Testing; I, intermediate or susceptible, increased exposure according to the applicable framework; IPM, imipenem; MEM, meropenem; MIC, minimum inhibitory concentration; NI, no applicable interpretation; NT, not tested or not reported; PTZ, piperacillin-tazobactam; R, resistant; S, susceptible; TMP-SMX, trimethoprim-sulfamethoxazole.
